# Comparison of Long-Term Survival of Patients with BCLC Stage B Hepatocellular Carcinoma after Liver Resection or Transarterial Chemoembolization

**DOI:** 10.1371/journal.pone.0068193

**Published:** 2013-07-09

**Authors:** Jian-Hong Zhong, Bang-De Xiang, Wen-Feng Gong, Yang Ke, Qin-Guo Mo, Liang Ma, Xing Liu, Le-Qun Li

**Affiliations:** 1 Hepatobiliary Surgery Department, Tumor Hospital of Guangxi Medical University, Nanning, People’s Republic of China; 2 Gastrointestinal Surgery Department, the People’s Hospital of Liuzhou, Liuzhou, People’s Republic of China; University of Navarra School of Medicine and Center for Applied Medical Research (CIMA), Spain

## Abstract

**Background and Aims:**

Treatment of patients with Barcelona Clinic Liver Cancer Stage B hepatocellular carcinoma (BCLC-B HCC) is controversial. This study compared the long-term survival of patients with BCLC-B HCC who received liver resection (LR) or transarterial chemoembolization (TACE).

**Methods:**

A total of 257 and 135 BCLC-B HCC patients undergoing LR and TACE, respectively, were retrospectively evaluated. Kaplan–Meier method was used for long-term survival analysis. Independent prognostic predictors were determined by the Cox proportional hazards model.

**Results:**

The hospital mortality rate was similar between groups (3.1% vs. 3.7%; *P* = 0.76). However, the LR group showed a significantly higher postoperative complication rate than the TACE group (28 vs. 18.5%; *P* = 0.04). At the same time, the LR group showed significantly higher overall survival rates (1 year, 84 vs. 69%; 3 years, 59 vs. 29%; 5 years, 37 vs. 14%; *P*<0.001). Moreover, similar results were observed in the propensity score model. Three independent prognostic factors were associated with worse overall survival: serum AFP level (≥400 ng/ml), serum ALT level, and TACE.

**Conclusions:**

LR appears to be as safe as TACE for patients with BCLC-B HCC, and it provides better long-term overall survival. However, prospective studies are needed to disclose if LR may be regarded as the preferred treatment for these patients as long as liver function is preserved.

## Introduction

Hepatocellular carcinoma (HCC) is associated with poor prognosis and its incidence is increasing in many countries [1], [2]. Our understanding of HCC pathogenesis and progression has advanced, and so have diagnostic methods, surgical techniques, and perioperative care. However, the prognosis of HCC patients remains discouraging because of the high postoperative recurrence rate and high cancer mortality. Identifying the optimal therapy for HCC patients is therefore important for maximizing their long-term survival.

Treatment outcomes for HCC patients are affected by multiple variables, including tumor burden, the Child-Pugh score of liver function reserve and the performance status of the patient [3]. Several staging systems have been described for HCC, including the Barcelona Clinic Liver Cancer (BCLC) classification [4] and Okuda [5], CLIP [6], and French [7] scores. Only the BCLC staging system takes into account the above three variables. It links staging with treatment indications and prognostic information, such as estimated life expectancy [8]. Studies have validated and proposed the clinical usefulness of this staging system [4], [9], making it one of the most reliable for HCC.

The BCLC intermediate stage, or stage B (BCLC-B), includes Child-Pugh A and B patients with large, single-focus HCC (>5 cm) and patients with multifocal HCC, defined as >3 tumors of any size, or 2–3 tumors with a maximal diameter >3 cm. To be categorized as BCLC-B, patients should be asymptomatic and have no vascular invasion or extrahepatic spread. The BCLC classification indicates that these patients are optimal candidates for transarterial chemoembolization (TACE) [4]. A systematic review of randomized clinical trials showed that TACE provided better 2-year survival than supportive treatment [10]. However, it is unclear whether TACE provides any long-term survival benefits. In addition, TACE has several potential disadvantages. Since the BCLC-B stage includes patients varying widely in tumor stage, hepatic function (Child-Pugh A or B) and disease etiology [11], [12], TACE may not be the optimal therapy for all of them. The technique also requires certain equipment and skills that may be lacking in some countries, or it may not be the first choice of physicians because of local surgical priorities and practices [13].

Liver resection (LR) is a simpler and less expensive alternative to TACE that may be more suitable for some HCC patients. Studies in North America, Europe and Asia concur that LR can be safely performed in patients with large or multinodular HCC who have good liver function [14], [15], [16], [17], [18]. The 5-year survival rate has been reported to be about 37% [14]. However, the indication of liver resection (LR) for BCLC-B HCC remains controversial and should be assessed in more studies [11]. It may be that BCLC selection criteria for LR are too restrictive, and that LR is a safe and effective therapy for a larger proportion of HCC patients. The aim of this study was to assess the therapeutic value of LR and compare it with TACE for treating BCLC-B HCC patients in southern China.

## Patients and Methods

### Ethics Statement

This study was approved by the institutional review board of Tumor Hospital of Guangxi Medical University. Written consent was given by the patients for their information to be stored in the hospital database and used for research.

### Patients

Retrospective analysis was carried out on medical records of patients diagnosed with HCC who were included in a prospective database of the Tumor Hospital of Guangxi Medical University from January 2000 to November 2007. Patients who received their initial HCC treatment at other centers were excluded. We excluded patients who received only local ablation therapy, systematic chemotherapy, sorafenib therapy or supportive care. Patients in whom a malignancy other than HCC had been diagnosed within a 5-year period before the initial HCC treatment were also excluded. The remaining patients were treated with either LR or TACE.

HCC diagnosis was confirmed after LR by histopathological examination of surgical samples in all patients. HCC diagnosis was confirmed in TACE patients by needle biopsy or by analysis of two images [ultrasonography, computed tomography (CT), or magnetic resonance imaging] in conjunction with a serum level of α-fetoprotein (AFP) higher than 400 ng/mL. Needle biopsy was performed in patients whose diagnosis based on imaging and AFP level was uncertain. Tumor status was assessed using various imaging techniques, including ultrasonography, CT scanning, magnetic resonance imaging, and/or hepatic angiography.

### Propensity Score Analysis

In order to reduce the bias in patient selection, a propensity score analysis was developed to investigate causal relationships between treatments and outcomes in a retrospective study other than a randomized controlled trial [19]. Clinical variables entered in the propensity model were age, gender, tumor size, tumor number, serum bilirubin, ALT, albumin, and AFP. Subsequently, a one-to-one match between the LR and TACE groups was obtained by using the nearest-neighbor matching method [20].

### Liver Resection

Only Child-Pugh A patients with BCLC-B disease were included in the current analysis. Indications for surgery were lack of ascites, hepatic encephalopathy, and hypersplenism, as well as the presence of appropriate residual liver volume, as determined by volumetric computed tomography [21]. Partial hepatectomy was performed following the techniques described by Zhou and coworkers [22]. The surgery started with a bilateral subcostal incision or L-shaped laparotomy with or without an upward midline extension. Intraoperative ultrasound was routinely performed to determine tumor location and assess the vascular anatomy of the liver. To minimize perioperative blood loss, Pringle’s maneuver was carried out intermittently, each time for less than 20 minutes, with a clamp-free interval of 5 min. The resection margin was more than 1 cm. Adequate drainage was monitored.

### TACE Procedure

Indications for using TACE for BCLC-B HCC patients were lack of ascites and main portal vein tumor thrombus, and presence of Child-Pugh A liver function. With the patient under local anesthesia, a 4F-to-5F French catheter was introduced into the abdominal aorta via the superficial femoral artery using the Seldinger technique. Hepatic arterial angiography was performed using fluoroscopy to guide the catheter into the celiac and superior mesenteric artery. Then the feeding arteries, tumor stain, and vascular anatomy surrounding the tumor were identified. A microcatheter was introduced through the catheter and directed to the feeding arteries. An emulsion of 5–15 ml lipiodol (Andre Guerbet, Aulnay-sous-Bois, France) and 5-fluorouracil (500 mg/m^2^) with or without adriamycin (30 mg/m^2^) was infused into the feeding arteries until blood flow had nearly stopped. A follow-up CT scan was arranged one month later to evaluate the effect of TACE. The course was repeated once every 1–2 months for 2–6 cycles.

The default treatment for patients was liver resection; patients were given TACE only upon request.

### Patient Follow-up

After surgery or TACE, all patients underwent regular follow-up involving a liver function test, measurement of serum AFP levels, abdominal ultrasonography, and chest radiography at an interval of 2–3 months in the first postoperative year and every 6 months in subsequent years. When intrahepatic recurrence was suspected, further investigation was carried out using CT and/or hepatic angiography. Chest CT or bone scans were conducted when distant metastasis was suspected. Biopsies were performed when necessary. Diagnoses of tumor recurrence and distant metastasis were based on cytohistology, or on the non-invasive diagnostic criteria for HCC used by the European Association for the Study of the Liver [23].

All recurrences and metastasis were evaluated for new treatment. Patients with recurrence were treated by hepatectomy, radiofrequency ablation therapy, microwave coagulation therapy, TACE, systemic chemotherapy, or sorafenib therapy. Therapy was decided based on extrahepatic disease, hepatic function, general health, and economic conditions.

### Study Endpoint

The primary endpoint of the study was survival time after LR and TACE. The survival time was defined as the time between the date of surgery or TACE and the date of death. Patients who were alive at the end of follow-up were censored.

### Statistical Analysis

Prior to this study, all demographic and clinicopathological data had been prospectively collected in a computer database. Differences between categorical data were analyzed using the chi-square test. Continuous data were expressed as mean ± SD. Differences between continuous data were analyzed using the *t* test. Survival analysis was calculated by the Kaplan–Meier method and group results were compared using the log-rank test. Multivariate analysis to identify independent prognostic factors was carried out using the Cox proportional hazards model to calculate the adjusted hazard ratio (HR) and 95% confidence interval (CI). All statistical analyses were performed with SPSS (version 16.0, Chicago, IL, USA). For all tests, a P value <0.05 was considered statistically significant.

## Results

### Study Population

During the study period, 2,758 consecutive southern Chinese patients with HCC were enrolled in the database. Of these, 913 (33.1%) were excluded because they had received their initial HCC treatment at other centers. Among the remaining 1,845 patients with complete survival data, 464 (25.1%) had BCLC-B disease without extrahepatic metastasis. Of these, we excluded 57 patients (12.3%) who received only local ablation therapy, systematic chemotherapy, sorafenib therapy or supportive care. Another 15 patients (3.2%) were excluded because they had been diagnosed with a malignancy other than HCC within a 5-year period before receiving initial HCC treatment. The remaining 392 patients (84.5%) were enrolled in the study.

Of these patients, 257 (65.6%) received LR and 135 (34.4%) received TACE. HCC diagnosis was confirmed after LR by histopathological examination of surgical samples. HCC diagnosis was confirmed in TACE patients by needle biopsy (11.6%) or using two imaging techniques in conjunction with a serum level of α-fetoprotein (AFP) higher than 400 ng/mL (88.4%). Needle biopsy was performed in 13 patients for whom diagnosis based on imaging and AFP was uncertain.

### Clinicopathological Data

Demographic and clinicopathological data for the 392 HCC patients are listed in Table 1. Most clinical characteristics were similar between the groups at baseline (Table 1). There were no significant differences in gender composition; tumor number; hepatitis incidence; levels of AFP, albumin, or alanine aminotransferase (ALT); prothrombin time; or hospital mortality. Patients in the TACE group were significantly older and had larger tumors and higher total serum bilirubin than those in the LR group. However, patients in the LR group had higher serum platelet counts, and they experienced more postoperative complications. The median age of all patients was <50 years. More than 90% of patients were male and HBsAg-positive. The proportion of hepatitis C virus infection was 2%.

**Table 1:** Preoperative clinicopathologic data of patients with Barcelona Clinic Liver Cancer stage B/Child-Pugh A hepatocellular carcinoma who received liver resection or transarterial chemoembolization (TACE).

Variable	Liver resection(n = 257)	TACE(n = 135)	*P* value
Age (yr)	46.8±12.0	48.7±12.5	0.08
Gender (M/F)	233 (90.7%)/24 (9.3%)	127 (94.1%)/8 (5.9%)	0.24
Tumor size, cm	8.9±3.0	8.8±2.5	0.63
Tumor number, S/M	199 (77%)/58 (23%)	104 (77%)/31 (23%)	0.93
Hepatitis B surface antigen	243 (94.6%)/14 (5.4%)	130 (90.4%)/5 (9.6%)	0.45
Hepatitis C antibody	5 (1.9%)	3 (2.2%)	0.85
α-fetoprotein			
≥400 ng/ml	143 (55.6%)	66 (48.9%)	0.20
< 400 ng/ml	114 (44.4%)	69 (51.1%)	
Albumin (g/dl)	3.9±5.6	3.8±4.7	0.07
Platelets (10^3^/µl)	193.4±74.9	185.7±79.4	0.34
Alanine aminotransferase (U/l)	56.5±64.7	62.4±45.5	0.35
Total bilirubin (umol/L)	14.5±5.3	15.1±8.5	0.40
Prothrombin time (s)	13.1±1.2	13.2±1.4	0.53
Esophageal varices			
Yes	46 (18%)	30 (22%)	0.30
No	211 (82%)	105 (78%)	
Hospital mortality	8 (3.1%)	5 (3.7%)	0.76
Postoperative complications	72 (28.0%)	25 (18.5%)	0.04
Median survival time (months)	42.9±26.1	21.0±18.8	<0.01

Values with “±” are written as mean ± SD.

Abbreviations: S/M, single tumor/multiple tumors.

### Mortality and Morbidity

The in-hospital mortality rate was similar in the LR group (3.1%) and the TACE group (3.7%). However, the postoperative complication rate was higher in the LR group (28%) than in the TACE group (18.5%; P = 0.04). The most common complication of LR was pulmonary infection (7%), while liver function failure (4.4%) was the most common complication of TACE. The specific complications of the two group patients are listed in Table 2.

**Table 2:** Postoperative complications in patients with Barcelona Clinic Liver Cancer stage B/Child-Pugh A hepatocellular carcinoma who received liver resection or transarterial chemoembolization.

Complication	No. (%) of patients	
	Liver resection(n = 257)	TACE(n = 135)
Pulmonary infection	18 (7.0)	4 (3.0)
Liver function failure	10 (3.9)	6 (4.4)
Pleural effusion	9 (3.5)	0 (0)
Abdominal infection	7 (2.7)	1 (0.7)
Incision dehiscence	6 (2.3)	0 (0)
Gastrointestinal hemorrhage	5 (1.9)	1 (0.7)
Wound infection	4 (1.6)	0 (0)
Postoperative abdominal bleeding	3 (1.2)	0 (0)
Deep venous thrombosis	3 (1.2)	2 (1.5)
Hepatic encephalopathy	2 (0.8)	1 (0.7)
Liver abscess	2 (0.8)	0 (0)
Intestinal obstruction	2 (0.8)	0 (0)
Pulmonary embolism	1 (0.4)	1 (0.7)
Puncture hematoma	0 (0)	6 (4.4)
Acute cholecystitis	0 (0)	3 (2.2)
Overall complications	72 (28)	25 (18.5)

### Survival Analysis

**Figure 1:**
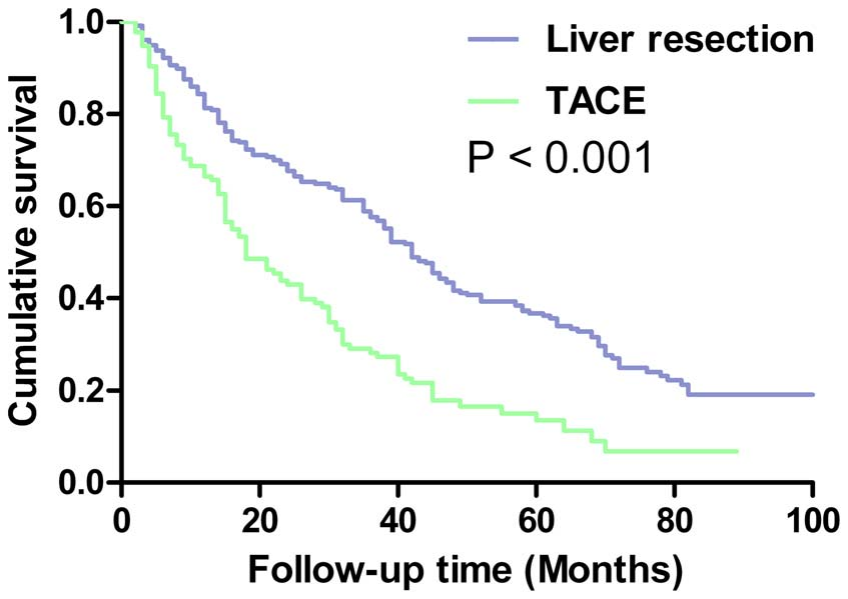
Overall survival curves of patients with BCLC-B HCC treated by liver resection or transarterial chemoembolization.

The overall survival rate was significantly better in the LR group than in the TACE group (P<0.05; Fig. 1). During a mean follow-up period of 29±14 months, 88 patients (34%) in the LR group and 73 patients (54%) in the TACE group died. The 1-, 3- and 5-year overall survival rates of patients in the LR group were 84%, 59%, and 37%, while the corresponding rates in the TACE group were 69%, 29%, and 14% (P<0.001). Median survival time was 42.9 months in the LR group and 21.0 months in the TACE group (P<0.001).

Several factors linked to survival were taken into account in the survival analysis (Table 3). Univariate analysis showed that tumor size, serum ALT level, serum AFP level (≥400 ng/ml), serum total bilirubin level, and treatment modalities were predictors of survival in the total study population. Multivariate analysis showed that serum AFP level ≥400 ng/ml (HR = 1.398, 95% CI 1.071–1.824, P = 0.014), serum ALT level (HR = 1.003, 95% CI 1.001–1.005, P = 0.027), and treatment modalities (HR = 2.149, 95% CI 1.592–2.902, P < 0.001) were predictors of survival (Table 3).

**Table 3:** Prognostic factors related to survival determined by univariate and multivariate analysis using the Cox proportional hazards model.

	Hazard ratio	95% CI	*P* value
**Univariate analysis**			
Gender	1.248	0.751–2.076	0.393
Age	0.993	0.982–1.004	0.214
Tumor size	1.084	1.038–1.138	<0.001
Tumor number	0.800	0.549–1.164	0.243
Hepatitis B virus infection	0.966	0.526–1.774	0.991
α-fetoprotein	1.426	1.098–1.851	0.008
Albumin	0.981	0.954–1.008	0.172
Platelets	0.999	0.998–1.001	0.443
Alanine aminotransferase	1.003	1.001–1.004	0.002
Total bilirubin	1.016	1.008–1.024	<0.001
Prothrombin time	1.042	0.946–1.148	0.404
Treatment modality	2.431	1.849–3.198	<0.001
**Multivariate analysis**			
Tumor size	1.029	0.981–1.079	0.245
α-fetoprotein	1.398	1.071–1.824	0.014
Alanine aminotransferase	1.003	1.001–1.005	0.027
Total bilirubin	1.009	0.999–1.019	0.064
Treatment modality	2.149	1.592–2.902	<0.001

### Survival Analysis of Patients with a Single Large Tumor

**Figure 2:**
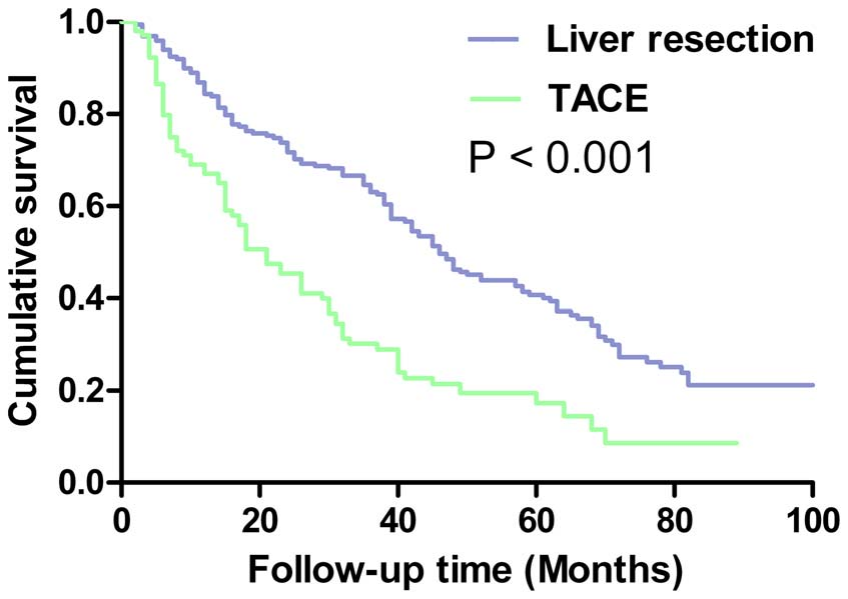
Overall survival curves of patients with a single large tumor of BCLC-B HCC treated by liver resection or transarterial chemoembolization.

The overall survival rate of patients with a single large tumor was significantly better in the LR group than in the TACE group (P<0.001; Fig. 2). The 1-, 3- and 5-year overall survival rates of patients after LR were 87%, 65%, and 41%; the corresponding rates for patients after TACE were 69%, 30%, and 18%.

### Survival Analysis of Patients with Multiple Tumors

**Figure 3:**
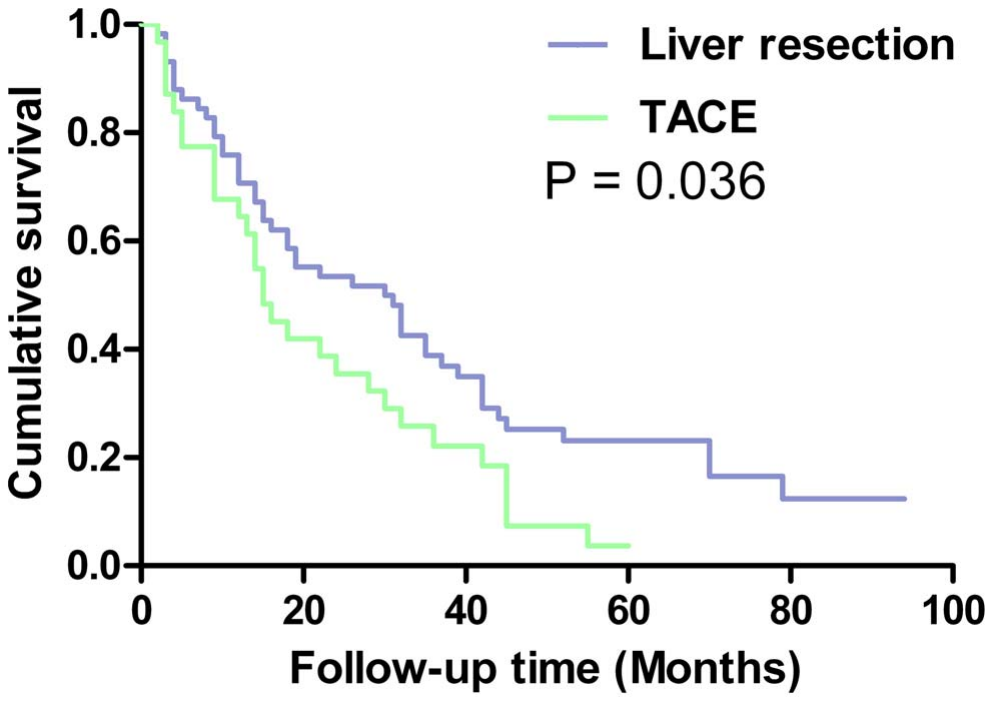
Overall survival curves of patients with multiple tumors of BCLC-B HCC treated by liver resection or transarterial chemoembolization.

The 1-, 3-, and 5-year overall survival rates for patients with multiple tumors were significantly better in the LR group (76%, 39%, and 24%) than in the TACE group (68%, 25%, and 4%) (P = 0.036; Fig. 3).

### Survival Analysis with Propensity Score

**Figure 4:**
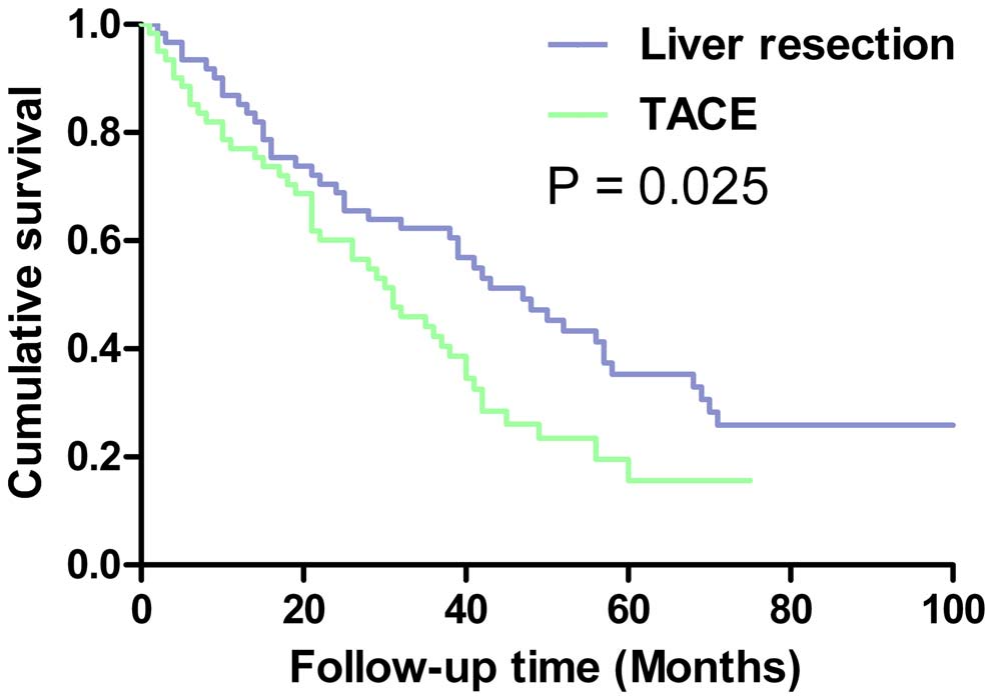
Overall survival curves of patients with BCLC-B HCC treated by liver resection or transarterial chemoembolization after propensity score.

Subsequently, for minimize the confounding factors, propensity score analysis with one-to-one nearest neighbor matching method was applied, including age, gender, tumor size and number, serum bilirubin, ALT, albumin, and AFP. Sixty-one pair patients were matched in each group. The above eight factors appeared to be well matched between groups. After matching, the overall survival rates of the LR group were also superior to that of the TACE group, with 87% vs. 77%, 62% vs. 44%, and 35% vs. 20%, respectively, for the 1-, 3- and 5-year overall survival rates (P = 0.025, Fig. 4).

## Discussion

This study shows that LR can offer better overall survival than TACE for selected BCLC-B HCC patients, and that both techniques offer acceptable morbidity and similar in-hospital mortality. Since large tumor size [24], [25], [26] and multiple nodules [25], [26], [27] are independent risk factors of recurrence and mortality [28], we analyzed overall survival for patients with large tumor size or multiple nodules independently. Results showed that LR can still offer better overall survival than TACE in both types of patients.

Our results in total population and propensity model analysis are consistent with recent reports that LR offers the best survival benefit for patients with BCLC-B HCC [14], [15], [24], [27], [29], [30], [31], [32], [33], [34], [35]. Therefore, large tumor size and multiple tumors should not exclude patients from LR. This surgery has also been shown to provide better quality of life than TACE for HCC patients [36].

The BCLC classification system has traditionally been used to guide treatment selection for patients with BCLC-B HCC. However, this system may not always provide the most appropriate recommendation. Surgical resection is the only hope for BCLC-B and -C HCC patients, but the BCLC classification considers these patients unsuitable for LR [37]. In recent decades, with advances in surgical techniques and preoperative preparation, the indications for LR have expanded. It is now considered a safe and routine surgical procedure and there is no absolute contraindication for it.

The BCLC staging classification recommends TACE as essentially the only option for patients with BCLC-B HCC and for some patients with BCLC-C HCC [4]. However, the BCLC-B stage includes a heterogeneous population of HCC patients, which means that not all patients will derive the same survival benefits from TACE [11]. In fact, the lack of a standard treatment methodology and patient selection criteria for TACE has made it difficult to draw any firm conclusions about the efficacy of the procedure for BCLC-B and -C HCC patients [11]. In addition, TACE can lead to drug resistance in some patients [38].

In order to compare the efficacy of LR for BCLC-B and C HCC reported by other recent studies, we address a comprehensive literature review on the subject (Table 4) [14], [15], [16], [24], [27], [29], [31], [32], [33], [34], [39], [40], [41], [42], [43], [44], [45], [46]. Our results show LR to offer better survival rates than TACE in the shorter and longer term (1 year, 84 vs. 69%; 3 years, 59 vs. 29%; 5 years, 37 vs. 14%; P<0.001). This is similar to a 3-year survival rate of 30% after TACE reported in other studies [24], [29]. In contrast, for patients with BCLC-B HCC who underwent LR, the median survival was 28 to 60 months, 3-year overall survival was 37–72%, and 5-year overall survival was 32–60%. Even for patients with BCLC-C HCC, the 5-year overall survival was more than 10% (Table 4). Actually, the recent study with large sample size by Torzilli and coworkers [46] collecting data from 10 centers showed that LR is in current practice widely. For patients with limited disease, there are no restrictions on tumor size, number or macrovascular invasion. Therefore, the BCLC selection criteria for LR seem too restrictive and should be expanded so that it can be considered a treatment option for patients with BCLC-B and -C HCC.

**Table 4:** Outcomes of liver resection for patients with BCLC-B or -C hepatocellular carcinoma in the recent literature (with sample size >50 and published between 2005 and 2013).

Study	Country	No. of patients	Disease characteristics	Median overall survival (mon)	Overall survival (%)	
					3-year	5-year
This study	Southern China	257	BCLC-B HCC	43	59	37
Chang 2012 [39]	Taiwan	478	BCLC-B and C HCC	–	52	41
Cheng 2012 [40]	Taiwan	104	Tumor size >5 cm	–	–	About 60
Cho 2007 [41]	South Korea	61	Tumor size: 5∼10	–	59	53
Delis 2010 [42]	Greece	66	Tumor size >5 cm	36	37	32
Ho 2009 [15]	Taiwan	122	BCLC-B HCC	42	52	37
Hsu 2012 [24]	Taiwan	268	BCLC-B/C/D HCC	–	63	43
Huang 2012 [43]	Taiwan	139	Tumor size >10 cm or adjacent organ invasion, or ruptured tumor	20	39	29
Ishizawa 2008 [16]	Japan	126	Multiple HCC with or without portal hypertention	–	72	58
Liau 2005 [44]	USA	82	Tumor size ≥10 cm	32	48	33
Lin 2010 [27]	Taiwan	93	BCLC-B HCC	28	49	–
Ng 2005 [31]	Asia, Europe and the USA	380	BCLC-B HCC	37	50	39
Ruzzenente 2009 [14]	Italy	105	Tumor size >5 cm with or without multinodular	14	–	16
Pawlik 2005 [33]	Asia, Europe and the USA	300	HCC ≥10 cm	20	37	27
Pawlik 2005 [34]	Asia, Europe and the USA	102	BCLC-C HCC	11	17	10
Torzilli 2013 [46]	Eastern & Western Experiences	663	BCLC-B HCC	–	71	57
		274	BCLC-C HCC	–	49	38
Wang 2008 [29]	Taiwan	243	BCLC-B HCC	60	64	51
Yamashita 2011 [32]	Japan	53	HCC ≥10 cm	–	43	35
Yang 2012 [45]	Eastern China	511	BCLC-C HCC	28	41	31

Our results, obtained with a population in southern China, are similar to those reported in studies in other Asian populations. Our data showed 3- and 5-year overall survival of LR patients of 59% and 37%, with a median survival time of 43 months. These results are similar to those reported by Wang and coworkers [29] for patients in Taiwan. In another study conducted in Taiwan, overall survival for patients with ≥2 tumors was higher after LR than after TACE [15]. A study by Hsu *et al*. [24] in Taiwan compared the efficacy of LR and TACE for patients with HCC beyond the Milan criteria. This study concluded that LR is as safe as TACE and provides better long-term survival for HCC patients beyond the Milan criteria [24]. In a retrospective study very similar to ours, Lin *et al*. [27] included 171 patients in Taiwan with BCLC-B/Child-Pugh A HCC. Those authors also found LR to be as safe as TACE and to provide better survival rates. The 3-year overall survival was 49% for the LR group and 2% for the TACE group (P<0.001) [27]. Taken together, these studies argue for considering LR as the preferred treatment for patients with BCLC-B HCC.

Several studies have examined prognostic factors for survival of BCLC-B HCC patients after LR [15], [24], [27], [31]. These studies reported different risk factors linked to long-term survival. Generally speaking, patients with no risk factors are predicted to have the best prognosis, while patients with three or more risk factors are predicted to have the worst overall survival [31]. In the present study, tumor size, ALT, AFP (≥400 ng/ml), total bilirubin, and TACE were associated with worse overall survival. However, on multivariate analysis, only AFP (≥400 ng/ml), ALT, and TACE were independent predictors of overall survival. Another study [47] also found AFP level to be an independent predictor of mortality in hepatitis C-related HCC. Though a few recent studies [48], [49] proposed that preoperative AFP levels are not useful for predicting postoperative survival of patients with early-stage HCC, AFP is used as a biomarker of HCC diagnosis [50] and has even proven useful as a marker for predicting antitumor response after radiofrequency ablation [51] and sorafenib therapy [52].

Like AFP, serum ALT was a predictor of survival in our BCLC-B HCC patients. This may reflect the fact that more than 90% of our HCC patients were HBV carriers; in such patients, long-term change in serum ALT is an independent predictor of risk for HCC [53]. At the same time, ALT is a widely used indicator of liver function and serum ALT level is associated with the degree of inflammation in liver disease [54]. Thus we may have detected ALT as a survival predictor because HCC patients with long-term HBV infection have worse prognosis.

There are several potential limitations of this study. First, most of our patients were males younger than 50 with chronic HBV infection. Therefore, the results may not be representative of all HCC patients. Second, this is a retrospective study, so there may have been selection bias. Nevertheless, all the patients in this study were admitted consecutively into our hospital for treatment. Moreover, the results from the propensity score analysis were similar to that from the overall population. Third, our results showing the superior results of LR compared to TACE should be used with discretion, since when tumor recurrence occurs or is likely, other therapies such as sorafenib, TACE or conservative treatment should be adopted.

In conclusion, LR yielded better overall survival than TACE in patients with BCLC-B HCC. For BCLC-B HCC patients with preserved liver function, LR may be considered the preferred treatment. However, prospective studies with large sample size are needed to reassess the efficacy of LR for these patients.
