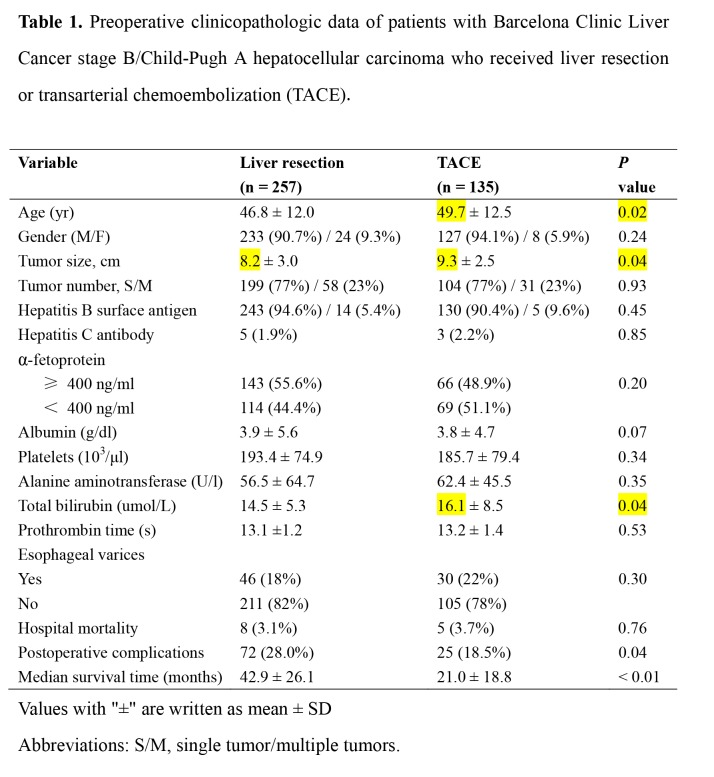# Correction: Comparison of Long-Term Survival of Patients with BCLC Stage B Hepatocellular Carcinoma after Liver Resection or Transarterial Chemoembolization

**DOI:** 10.1371/annotation/32a8c7e3-5efc-478b-b374-c8cbfd1d3968

**Published:** 2014-01-21

**Authors:** Jian-Hong Zhong, Bang-De Xiang, Wen-Feng Gong, Yang Ke, Qin-Guo Mo, Liang Ma, Xing Liu, Le-Qun Li

There are several errors in the data listed in Table 1. Please see the correct table with highlighted changes: 

**Figure:**